# Static and Dynamic Experimental Analysis of the Galloping Stability of Porous H-Section Beams

**DOI:** 10.1155/2014/746826

**Published:** 2014-08-28

**Authors:** F. Gandia, J. Meseguer, A. Sanz-Andrés

**Affiliations:** ^1^E.U.I.T. Aeronáutica, Universidad Politécnica de Madrid, Plaza Cardenal Cisneros, No. 3, 28040 Madrid, Spain; ^2^IDR/UPM, Universidad Politécnica de Madrid, Plaza Cardenal Cisneros, No. 3, 28040 Madrid, Spain

## Abstract

The phenomenon of self-induced vibrations of prismatic beams in a cross-flow has been studied for decades, but it is still of great interest due to their important effects in many different industrial applications. This paper presents the experimental study developed on a prismatic beam with H-section. The aim of this analysis is to add some additional insight into the behaviour of the flow around this type of bodies, in order to reduce galloping and even to avoid it. The influence of some relevant geometrical parameters that define the H-section on the translational galloping behaviour of these beams has been analysed. Wind loads coefficients have been measured through static wind tunnel tests and the Den Hartog criterion applied to elucidate the influence of geometrical parameters on the galloping properties of the bodies under consideration. These results have been completed with surface pressure distribution measurements and, besides, dynamic tests have been also performed to verify the static criterion. Finally, the morphology of the flow past the tested bodies has been visualised by using smoke visualization techniques. Since the rectangular section beam is a limiting case of the H-section configuration, the results here obtained are compared with the ones published in the literature concerning rectangular configurations; the agreement is satisfactory.

## 1. Introduction

It is well known that two-dimensional bluff bodies in a cross-flow are subject to typical aeroelastic phenomena like vortex shedding, galloping, flutter, and buffeting. Some of these phenomena can even appear coupled occasionally. Galloping is a typical instability of flexible, lightly damped structures. Under certain conditions these structures may have large amplitude, normal to wind oscillations, at much lower frequencies than those of vortex shedding found in the Kármán vortex street. Although many two-dimensional bodies can experience galloping episodes, this kind of instability seems to appear more rather in bluff bodies than in streamlined ones.

**Figure 1:**
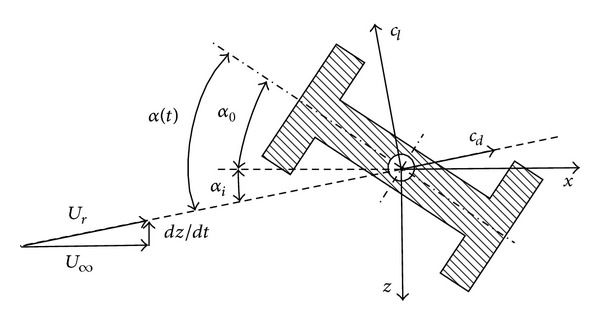
Sketch of a typical H beam. *U*_*∞*_ is the unperturbed upstream flow velocity, *dz*/*dt* is the vertical velocity due to transversal body oscillation, *α* is the angle of attack of the body under static conditions, and *α*(*t*) is the actual one. Lift and drag coefficients are *c*_*l*_ and *c*_*d*_, respectively.

Theoretical foundations of galloping are well established and can be easily understood through an extremely simple theory like the one of Den Hartog [1], which, in a first attempt, is enough to elucidate if a given two-dimensional body can gallop or not. According to Den Hartog, galloping can be explained by taking into account that, even if the incident wind velocity *U*_*∞*_ is uniform and constant, in a body reference frame the lateral oscillation of the body can cause the total velocity to experience changes both in its magnitude and direction with time. Therefore, the body angle of attack also changes with time and hence the aerodynamic forces acting on it (Figure 1).

**Figure 2:**
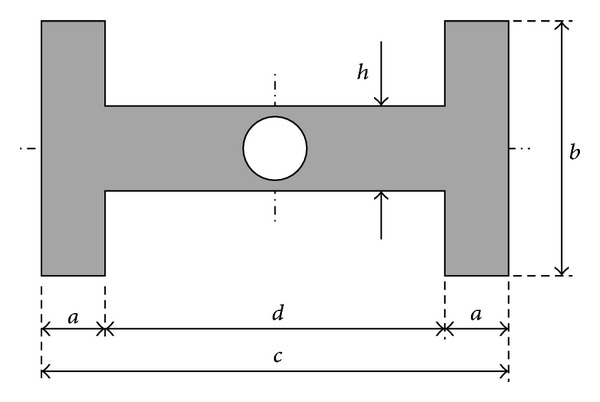
Parameters that define the geometry of a typical H beam.

Concerning the stability analysis, it is based on the simplest model of galloping (one degree of freedom); it is assumed that a two-dimensional body, whose mass per unit length is *m*, is elastically mounted on a support with a damping coefficient *ζ* and a stiffness *mω* ^2^ (where *ω* is the angular natural frequency). Within this approximation, if the aerodynamic force (proportional in this case to *dz*/*dt*) is considered as a contribution to the total damping of the system, the total damping coefficient is

(1) $$\begin{array}{c} \zeta_{T}=\zeta +\frac{\rho U_{\infty }b}{4m\omega }\left(\frac{dc_{l}}{d\alpha }+c_{d}\right), \end{array}$$

where *U*_*∞*_ stands for the upstream flow velocity and *b* for a transversal characteristic length of the body (Figure 2). Therefore, the oscillation will be damped if *ζ*_*T*_ > 0 and unstable if *ζ*_*T*_ < 0. As the mechanical damping *ζ* is generally positive, instability will only occur if the parameter *H* = *dc*_*l*_/*dα* + *c*_*d*_ < 0, expression known as Den Hartog criterion, which is a necessary condition for galloping instability. The sufficient condition for galloping is *ζ*_*T*_ < 0, or, according to (1) and the above definition of the parameter *H*, *H* < −4 *mζω*/(*ρU*_*∞*_ *b*). Note that in this last expression the second member tends to be zero when the wind velocity increases, which means that the possibility of galloping becomes higher as the wind velocity increases.

From inspection of (1), since the drag coefficient is positive, it is clear that the slope of the lift coefficient versus angle of attack curve must be negative, which means that the body must be stalled (*dc*_*l*_(*α*)/*dα* < 0) and that the absolute value of this slope curve must be larger than the drag coefficient.

Galloping has focused the attention of many researchers during the last decades because of its impact in very common problems related to ice accretion on electric transmission lines, traffic signal gantries and structures, catenary leads, and many other configurations. In the case of bridges, some situations are well described, where, without reaching the collapse of the structure, large oscillations have occurred in some elements, as in the case of Commodore Barry Bridge over the Delaware River in USA, in 1973, or more recently the case of Dongping Bridge in China, which in 2006 resulted in partial ruptures of structural anchoring elements. It may be useful to recall here that the collapse of the Tacoma Narrows Bridge in 1940, one of the greatest disasters in history that occurred on a bridge, was not due to the phenomenon of galloping but to flutter, which has a different aeroelastic origin.

A fairly large number of papers dealing with the galloping properties of a wide spectrum of geometries have been published (reviews on different bluff bodies can be found in [1–4]). It must be pointed out that most of the effort in galloping research has been concentrated in bodies with square or rectangular cross-sections [5, 6], although prismatic bodies with other cross-sectional shapes have been also considered: triangular cross-section bodies [7–9], biconvex or rhomboidal cross-sections [10], elliptical cross- sections [11], and even H cross-section beams [12–16]. In the last years, some research on galloping has been carried out at IDR/UPM, and a systematic parametric analysis of simple cross-section two-dimensional bodies has been accomplished [7–11, 16].

In this paper, the transverse galloping characteristics of H shaped beams are analysed through static tests (measuring global aerodynamic forces and the pressure distributions on the surfaces of the models) and dynamic tests (allowing the models to freely oscillate when subjected to a uniform flow). The aim of this study is to elucidate how the body geometry (Figure 2) affects the galloping characteristics and the analysis of suitable geometry modifications to suppress galloping phenomena, by using lateral porous plates instead of solid ones.

In all cases, the H-section considered is inscribed in a rectangle with a ratio *c*/*b* = 2 (see Figure 2), being a section widely used in civil construction as in the case of some bridge decks or as vertical suspension bars. It should be noted that, as has been studied by some authors [17], the rectangles falling into the relationship 0.5 < *c*/*b* < 3 show galloping in uniform flow conditions and for a wide range of turbulent flows.

## 2. Experimental Setup and Procedures

### 2.1. Static Tests

Experiments concerning static tests were carried out at the Laboratorio de Aerodinámica, E.U.I.T. Aeronáutica, Universidad Politécnica de Madrid. An open return Plint & Partners modified wind tunnel was used. This wind tunnel has a 8 : 1 contraction ratio and a rectangular test chamber 0.160 m wide, 1.2 m high, and 1.50 m long. The speed in the test section can be up to 30 m/s, and the turbulence intensity is 0.7%. The nonuniformity of the flow at the test chamber, (*U*_max⁡_ − *U*_min⁡_)/*U*_mean_, is less than 1%, so that this wind tunnel becomes appropriate for low Reynolds number tests [18]. In the above expression *U* stands for the velocity, and the subscripts max, min, and mean indicate maximum, minimum, and average, respectively.

For the aerodynamic forces measurement, an external, pyramidal, three-component, electronic Plint Ltd. balance was used, which allows the lift and drag force to be measured, as well as the pitching moment of the body placed inside the test chamber. Measurements require the subtraction of the initial values and the results are then multiplied by the calibration constants of all load cells.

Dynamic pressure inside the test chamber is measured by a standard pitot tube attached to the top wall of the wind tunnel, just ahead of the model, and connected to an MP6KS Air Ltd. pressure transducer. From pitot tube measurement, taking into account the temperature and ambient pressure at the laboratory, the air flow velocity *U*_*∞*_ is obtained, leading to a Reynolds number, *Re* = *U*_*∞*_ *c*/*ν*≅10^5^, where *c* stands for the body chord, as defined in Figure 2, and *ν* for the kinematic viscosity of air.

All the electrical signals coming from the wind tunnel are acquired by a HP 6110 laptop, through National Instruments DAQ Card-6062 E with 16 analogical input channels.

Before a tests campaign, some previous tests were made, both at low and high angles of attack, to determine the optimum sampling frequency rate and the sampling time.

The different models were made of Necuron resin and machined in a Roland MDX-540 milling machine with a 0.1 mm precision. All of them are of 158 mm span, thus leaving a 1 mm gap between the wind tunnel walls and the lateral surfaces of the models. It must be pointed out that this gap does not affect the two-dimensional behaviour of the model; the reasons are that these gaps are very narrow and they are placed at the boundary layers that develop at the wind tunnel walls [19]. Bodies are fixed to the balance through a 12 mm steel rod, placed at the centre of mass of the models, as sketched in Figure 1.

Furthermore, some visualization tests were performed by using a small smoke wind tunnel (the working section is 0.4 m high, 0.04 m wide, and 0.6 m long), in order to get additional information on the morphology of the flow past the models.

In experiments, the lift, *l*(*α*), drag, *d*(*α*), and pitching moment, *m*(*α*), were measured at angles of attack varying from *α* = 0° to *α* = 90° at variable Δ*α* step (this step is smaller, Δ*α* = 1°, where the lift slope curve is negative and galloping can occur, and larger, Δ*α* = 5°, where the lift slope is positive). The angle of attack can be set with ±0.5° accuracy. From measured results the aerodynamic coefficients are determined, *c*_*l*_(*α*) = *l*(*α*)/(*q*_*∞*_ *c*), *c*_*d*_(*α*) = *d*(*α*)/(*q*_*∞*_ *c*), and *c*_*m*_(*α*) = *m*(*α*)/(*q*_*∞*_ *c* ^2^), and then the Den Hartog parameter *H* = *dc*_*l*_/*dα* + *c*_*d*_ is calculated. Overall uncertainty in the freestream velocity is estimated to be 0.25% and in the force coefficients 2.1%.

### 2.2. Dynamic Tests

The dynamic tests were carried out at IDR/UPM. An open return wind tunnel, A4C, has been used [20]. A4C wind tunnel has a rectangular test section 0.2 m wide, 1.8 m high, and 2 m long. The turbulence intensity at the tests section is under 3%, and the nonuniformity of the flow is less than 2%. The models chord is *c* = 0.2 m and the height *b* = 0.1 m. Models span is 0.196 m.

**Figure 3:**
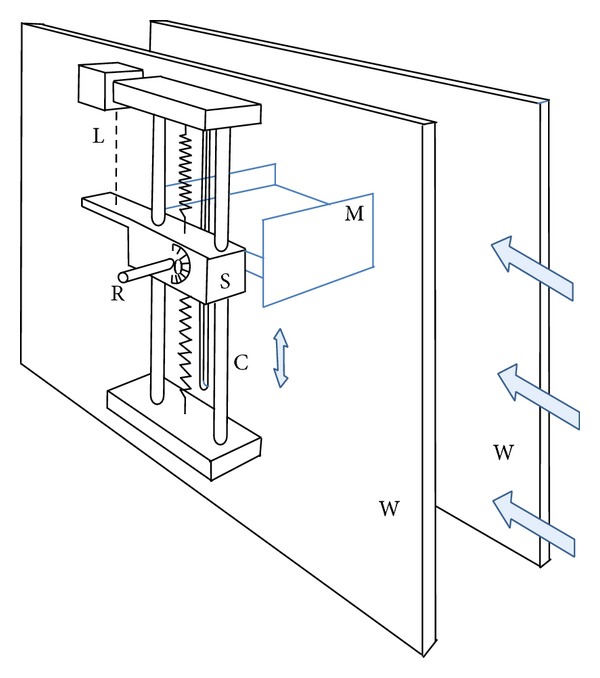
General view of the dynamic test device. Wind tunnel walls (W), model (M), rod (R), sliding support (S), columns (C), and laser displacement sensor (L).

As sketched in Figure 3, the model M, located inside the wind tunnel test chamber, between the wind tunnel walls W, is anchored to the sliding support S through the rod R. The support S can move vertically along the two steel columns C, so that to allow the vertical displacement of the rod there is a vertical slot in the corresponding test chamber wall. The support device is equipped with air lubricated bushings in order to reduce mechanical friction as much as possible. Two springs limit the vertical amplitude of the oscillation movement of the model. These springs are interchangeable to adjust the stiffness of the system, thus allowing fixing the onset velocity of galloping within the wind tunnel velocities range.

The rod R is attached in such a way that the angle of attack of the model can be set from 0° to 90° with ±0.5° accuracy. The vertical displacement is measured with a laser sensor L (MEL model M7L100) with 100 mm measuring range and 64 *μ*m resolution. As already said, the model, as well as part of the rod, is located inside the wind tunnel test chamber, whereas the oscillation mechanism and instrumentation are outside the test chamber; all this external equipment is enclosed a tight box which is kept at the test chamber pressure because of the vertical slot.

Dynamic pressure inside the test chamber is measured by a pitot tube (Air Flow model 048) attached to the ceiling of the wind tunnel, connected to a P-3061-2WD, Schaevitz Lucas pressure transducer. In each test, the velocity of the wind tunnel was varied from 0.5 to 22 m/s, with increments of 0.5 m/s near the critical velocity and 1 m/s in the rest of the measurement range. In this case, the overall uncertainty in the freestream velocity is estimated to be 0.3%.

Once the stiffness of the system is experimentally measured, the angular natural frequency *ω* and the structural damping *ζ* are determined letting the model vibrate freely at zero wind speed. By comparing the residence time of a fluid particle, *t*_*r*_~*c*/*U*_*∞*_, with the characteristic time, *t*_*o*_~1/*ω*, the condition for almost static criterion is obtained; that is, *t*_*r*_ ≪ *t*_*o*_, or *U*_*∞*_ ≫ *cω*.

## 3. Experimental Results 

### 3.1. Static Tests

In the case of static tests, two types of H-section beam configurations were considered, although in all tested configurations the lengths *c* and *b* were kept constant (*c*/*b* = 2; see Figure 2). In all cases, the H beam chord, *c*, was kept constant, *c* = 0.1 m, but, in type A configurations, the length *a* was varied from 2*a*/*c* = 0.03 to 2*a*/*c* = 1.0 (note that, according to Figure 2, since (2*a* + *d*)/*c* = 1, the value 2*a*/*c* = 1.0 gives *d* = 0, which corresponds to a rectangular beam). The aim of this set of tests was to study the influence of the thickness of the extreme vertical plates on the galloping behaviour.

In the second set of H beam configurations, type B, the thickness of the vertical plates *a* was kept constant (2*a*/*c* = 0.03) as well as the overall dimensions of the H beam (*c*/*b* = 2), but circular holes of different diameters were drilled on the vertical plates to analyse the influence of its porosity on the galloping response. The porosity is defined as the ratio to the whole surface of the drilled holes made on the vertical plates, that is, *nπr* ^2^, where *n* is the number of holes made in the plates and *r* the hole radius, to the surface of the vertical plate which can be perforated, (*b* − *h*)*s*, where *b* and *h* are defined in Figure 2 and *s* stands for the model span. Thence the porosity *ϕ* becomes *ϕ* = *nπr* ^2^/[(*b* − *h*)*s*]. The holes were uniformly distributed in two different rows on each vertical surface.

**Figure 4:**
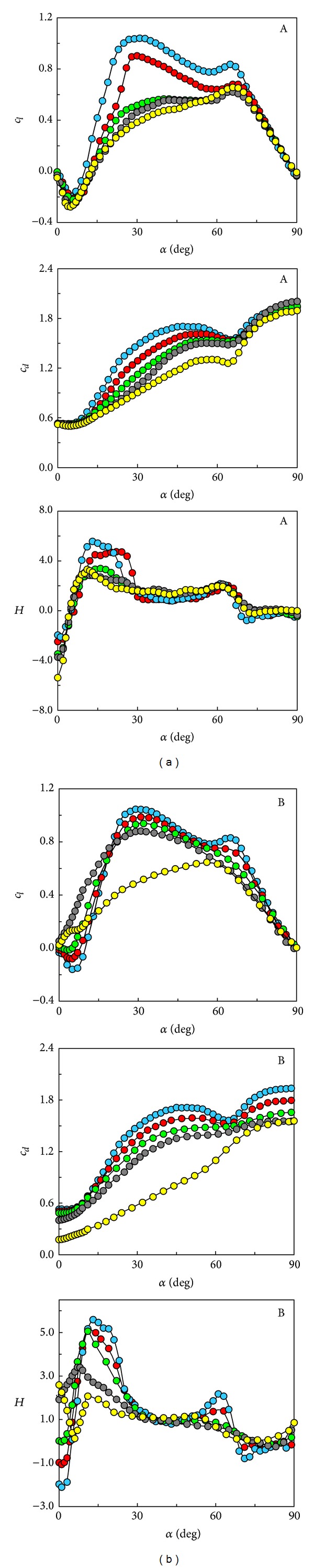
Variation with the angle of attack, *α*, of the lift coefficient, *c*_*l*_, the drag coefficient, *c*_*d*_, and the Den Hartog parameter *H*. Left column, type A beams, with different 2*a*/*c* ratio (the symbols identify the values of the parameter 2*a*/*c* according to the key: 2*a*/*c* = 1, yellow; 2*a*/*c* = 0.8, grey; 2*a*/*c* = 0.6, green; 2*a*/*c* = 0.2, red; 2*a*/*c* = 0.03, blue). Right column, type B beams, with vertical plates with different porosities (the symbols identify the values of the parameter *ϕ* according to the key: *ϕ* = 1, yellow; *ϕ* = 0.6, grey; *ϕ* = 0.4, green; *ϕ* = 0.2, red; *ϕ* = 0, blue).

The results obtained with type A beams are depicted in the left column of Figure 4, whereas those corresponding to porous H beams (type B) are shown in the right column of the same figure.

Concerning type I beams, the results show that the lift slope becomes negatively close to *α* = 0° until it reaches a minimum at *α*≅6° (the beam is stalled); this behaviour is the same independently of the value of the parameter 2*a*/*c*; beyond this minimum the lift coefficient starts to grow as the angle of attack grows, so that the lift coefficient slope curve becomes positive. From the point of view of galloping, there is another region, close to *α* = 90°, where the lift slope becomes again largely negative.

The drag coefficient increases as the angle of attack grows in almost all the whole range (0° ≤ *α* ≤ 90°)except close to *α*≅65° where relative minima appear no matter the value of the parameter 2*a*/*c* is. These local minima coincide with the angles of attack where the lift slopes start to be almost constant and largely negative (Figure 4).

**Figure 5:**
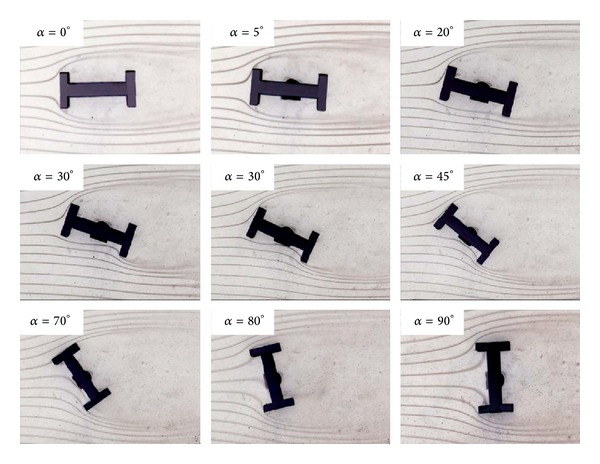
Smoke visualization of the flow past an H beam with geometrical parameters 2*a*/*c* = 0.25 and *b*/*c* = 0.45; note that the colours are inverted to enhance visualization according to Gandía et al. [16].

To get some additional insight into this behaviour, some visualization in a small smoke wind tunnel was performed, and some of the pictures obtained are shown in Figure 5. Note that for *α* < 55° the upper boundary layer separates at the upper leeward corner of the H beam, whereas for *α* > 75° the separation takes place at the upper windward corner. For *α*≅65° there is a smoke streamline which is almost parallel to the H beam upper surface, which probably implies a narrow wake behind the body at these values of the angle of attack.

**Figure 6:**
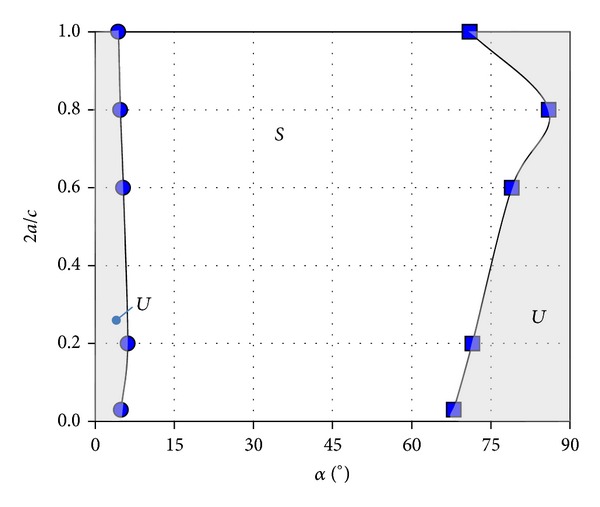
Stability diagram of H-section beams in the H geometry versus angle of attack plane (2*a*/*c* versus *α* plane), where the lengths *a* and *c* are defined in Figure 2. Shadowed areas indicate unstable regions, although the right hand side region is only marginally unstable according to Gandía et al. [16].

With the experimental measurements *c*_*l*_ and *c*_*d*_, the Den Hartog function *H* = *dc*_*l*_/*dα* + *c*_*d*_ has been determined and represented in Figure 4. As can be observed, there is a region close to *α* = 0° where H beam configurations are unstable (see also Figure 6), and there is another region close to *α* = 90° where these bodies are weakly unstable.

Between these two regions H beams are not prone to transversal galloping oscillations. It must be remarked that, for large values of the angle of attack, although according to Den Hartog criterion the H function is negative, the absolute values of this parameter are so small so that the resulting motions are only marginally unstable.

Note that the size of this region decreases as the parameter *a* grows, until a given critical value of this parameter is reached. The size of the unstable region increases as the parameter *a* grows beyond the critical value. Note also that the unstable region must be almost the same for 2*a*/*c* = 0 and 2*a*/*c* = 1, provided the chord *c* is large enough (in both cases the H beam behaves as a rectangular cross-section body).

**Figure 7:**
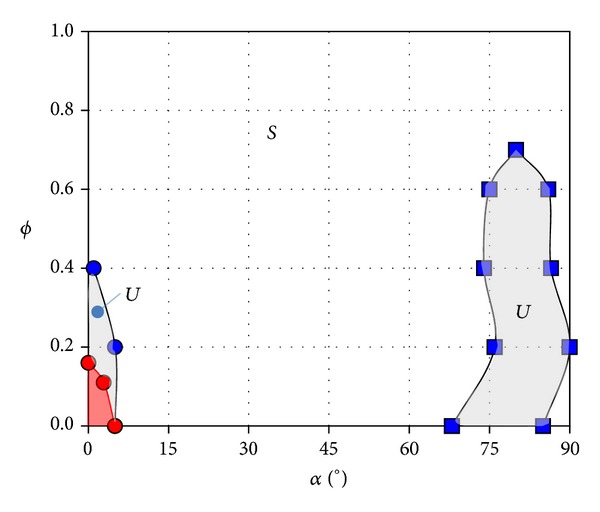
Stability diagram of H beams in the porosity versus angle of attack plane (*ϕ* versus *α* plane). The results correspond to H beams with 2*a*/*c* = 0.03 and *c*/*b* = 2, where the lengths *a*, *b*, and *c* are defined in Figure 2. Shadowed areas indicate unstable regions, although the right hand side region is only marginally unstable. Note that, for large values of the porosity, H beams are not unstable. Red symbols correspond to dynamic test results (see Section 3.2).

The differences between the tested type B beams are in the porosity *ϕ* of the vertical plates, which was changed from *ϕ* = 0 (solid vertical plates, yellow symbols in the right column of Figure 4) to *ϕ* = 1 (no vertical plates, blue symbols). The measured results, *c*_*l*_, *c*_*d*_, and *H* versus *ϕ*, are shown in Figure 4. According to these plots, it seems that H beams are stable for large enough values of the porosity, both for small and large values of the angle of attack (Figure 7).

The absence of gallop for large porosities, say, greater than *ϕ* = 0.4, can be explained by the fact that the central core of the H-section, without the vertical plates, is a rectangular section with *h*/*c* = 0.25 (see Figure 2) which as reported elsewhere is not prone to gallop [4, 21]. The measured results for type B beams show a behaviour similar to type A with 2*a*/*c* = 0.03, but, as the porosity tends to be 1, the curves progressively approach the behaviour of rectangular section with *h*/*c* = 0.25.

To better quantify the effects of porosity, static tests were carried out to obtain the pressure distribution in the core surface of the H beam (the central rectangular box).

**Figure 8:**
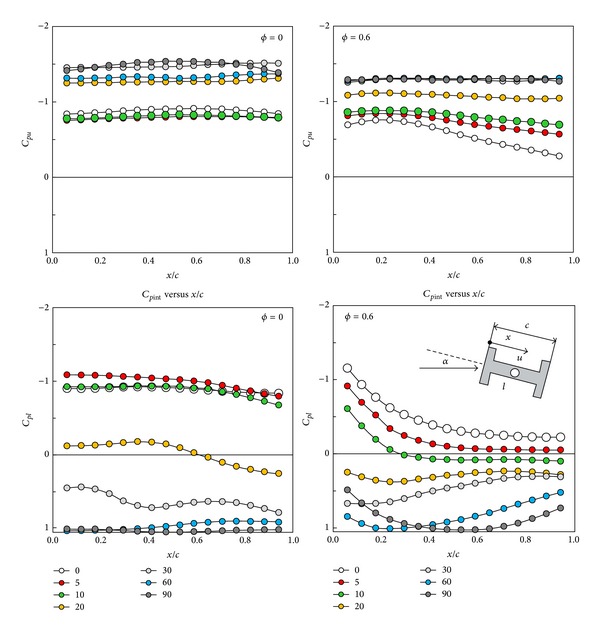
Pressure distributions on the surfaces of the central box of H-section beams. Results on the left column correspond to solid vertical walls, *ϕ* = 0, and those of the right column to a porosity *ϕ* = 0.6. Symbols identify the angle of attack according to the keys included in the inserts.

The results obtained for various angles of attack, for porosities *ϕ* = 0 and *ϕ* = 0.6, are shown in Figure 8. In both cases it was found that, starting from zero angle of attack, a moderate increase of angle of attack involves a progressive increase of the suction in the upper surface of the central rectangular box, while the suction decreases in the lower surface.

For zero porosity and angles of attack from 0° to 10°, there is a relative strong suction pressure in the lower surface that results in galloping. For porosity *ϕ* = 0.6 and the same values of the angle of attack, there is less suction pressure and hence gallop cannot occur. The study of the pressure distributions for intermediate porosities, *ϕ* = 0.2 and *ϕ* = 0.4 (not shown in Figure 8) at these low angles, shows that this is a general result: an increase of porosity involves a decrease of the suction pressure in the lower surface reducing the possibility of galloping. At angles of attack near to 90° galloping does not clearly appear in this type of tests.

All these results agree with those obtained by directly measuring the forces, with the wind tunnel balance, and included in the stability diagrams of Figures 6 and 7.

### 3.2. Dynamic Tests

**Figure 9:**
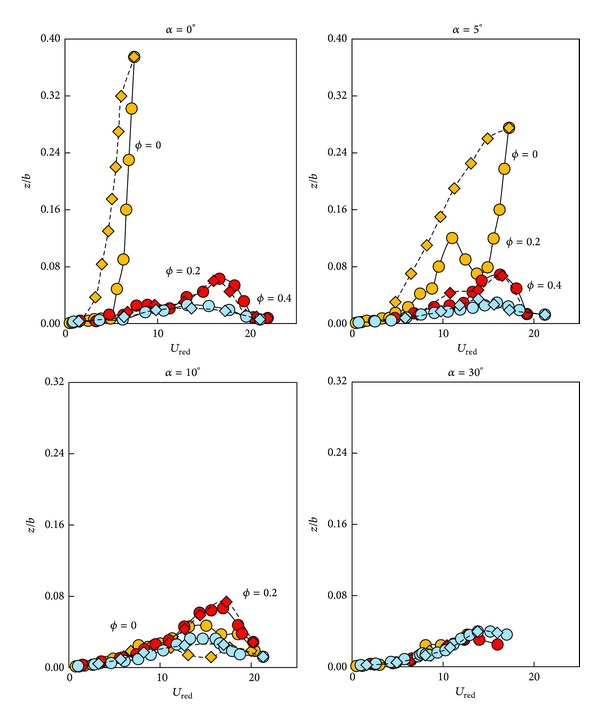
Variation of the root mean square of the dimensionless maximum oscillation amplitude, *z*/*b*, with the reduced speed, *U*_red_ = *U*_*∞*_/(*ωb*), at zero angle of attack, for three values of the porosity *ϕ*. Circles correspond to test series where the reduced velocity increases, whereas rhombi indicate decreasing reduced velocity.

The second set of H beam configurations, type B, with *c*/*b* = 2, was selected (now 2*a*/*c* = 0.05), with the objective of verifying the Den Hartog criterion for values of porosities 0, 0.2, and 0.4 and for angles of attack varying from 0 to 90°.  Figure 9 shows the root mean square (rms) of the maxima of the dimensionless vertical amplitude, *z*/*b*, where *z* stands for the vertical amplitude and *b* for the frontal height of the H-section beam, as a function of the reduced velocity, *U*_red_ = *U*_*∞*_/(*ωb*), for zero angle of attack.

Note that, at this angle of attack, gallop appears only when *ϕ* = 0. For higher porosities the prism has only a small amplitude oscillation. Note also the hysteresis region that appears in *ϕ* = 0 configurations (additional details on the hysteresis of the response of galloping bodies can be found in [4, 9, 22, 23]).

The results obtained for the three porosities, varying the angles of incidence from 0° to 90°, are also depicted in Figure 7. It can be seen that, for angles of attack close to zero degrees, the instability region is substantially reduced, and the galloping phenomenon disappears provided the porosity becomes larger than 0.1. Close to 90°, galloping instability completely disappears when dynamic tests are considered, at least in the range of wind speeds of experiments.

The reduction in the size of the instability region shown in Figure 7 can be explained because of the mechanical dissipation involved in an oscillation mechanism (even if air bushings are used) and of the higher turbulence existing in the flow of the wind tunnel used in the dynamic tests when compared with one existing in the wind tunnel used in the static tests. It is well known that for rectangular sections the turbulence increases the mixing in the shear layer of the separated region, reducing the suction peak in the lower surface at low angle of attack [2, 17, 24, 25]. This causes a reduction in the galloping effects and for higher values of turbulence the galloping is eliminated.

## 4. Conclusions

In this paper results obtained from an experimental campaign to analyse the influence of several geometric parameters on the galloping behaviour of H cross-section beams are presented. In this experimental campaign several wind tunnels have been used. A first wind tunnel was used to measure the aerodynamic coefficients of lift, *c*_*l*_, and drag, *c*_*d*_, at angles of attack from 0° to 90°, and then the Den Hartog criterion was applied. In order to improve the understanding of the physical behaviour of the air flow around the section, additional visualization tests were performed in a small smoke wind tunnel, and measurements of surface pressure distributions on the central box of an H-section beam were also carried out by using the former wind tunnel. Finally, dynamics tests in a third wind tunnel have been performed in order to verify the accuracy of the results provided by the Den Hartog criterion.

Although the analysis has been constrained to a few geometrical configurations, experimental results for the static tests show that, for the configuration under study, the influence of the parameter 2*a*/*c* does not substantially affect the phenomenon of galloping. On the other hand, porosity *ϕ* seems to be an important parameter that may effectively control the galloping behaviour. In fact, increasing the porosity from zero to 0.4, the behaviour progressively changes from the section with *c*/*b* = 2, which gallops close to 0°, to the section *h*/*c* = 0.25 that does not gallop at any angle of attack.

The results of the dynamic tests agree with the static ones, but they show that the region of unstable configurations in the *ϕ* versus *α* plane is smaller than the one obtained when the static Den Hartog criterion is applied, as one could expect taking into account previous results concerning the galloping behaviour of triangular cross-section bodies published elsewhere [7].

For this type of H-sections, a noticeable effect of the stream turbulence has been found. In this sense, the turbulence effect on the analysed H-section bodies seems to behave in a similar way as in rectangular section bodies.

It should be pointed out that hysteresis appears in the galloping of H-section beams, although it seems that the hysteresis phenomenon is restricted to H bodies with solid vertical surfaces.

As a brief summary, for the geometry studied (*c*/*b* = 2) and within the range of speed analyzed, the static and dynamic results show that, for H-section beams, the most critical angles of attack are close to *α* = 0° and, more weakly, close to 90°. The galloping behaviour seems not to be affected by the relative thickness (2*a*/*c*), but the porosity in the vertical plates seems to be an effective mechanism for controlling its appearance.
